# Effect of Season of Birth on Hippocampus Volume in a Transdiagnostic Sample of Patients With Depression and Schizophrenia

**DOI:** 10.3389/fnhum.2022.877461

**Published:** 2022-06-13

**Authors:** Nora Schaub, Nina Ammann, Frauke Conring, Thomas Müller, Andrea Federspiel, Roland Wiest, Robert Hoepner, Katharina Stegmayer, Sebastian Walther

**Affiliations:** ^1^Translational Research Center, University Hospital of Psychiatry and Psychotherapy, Bern, Switzerland; ^2^Support Center of Advanced Neuroimaging (SCAN), Inselspital, University Institute of Diagnostic and Interventional Neuroradiology, Bern, Switzerland; ^3^Department of Neurology, Inselspital, University Hospital and University of Bern, Bern, Switzerland

**Keywords:** depression, schizophrenia, season of birth, structural neuroimaging, hippocampus, gray matter

## Abstract

Psychiatric disorders share an excess of seasonal birth in winter and spring, suggesting an increase of neurodevelopmental risks. Evidence suggests season of birth can serve as a proxy of harmful environmental factors. Given that prenatal exposure of these factors may trigger pathologic processes in the neurodevelopment, they may consequently lead to brain volume alterations. Here we tested the effects of season of birth on gray matter volume in a transdiagnostic sample of patients with schizophrenia and depression compared to healthy controls (*n* = 192). We found a significant effect of season of birth on gray matter volume with reduced right hippocampal volume in summer-born compared to winter-born patients with depression. In addition, the volume of the right hippocampus was reduced independent from season of birth in schizophrenia. Our results support the potential impact of season of birth on hippocampal volume in depression.

## Introduction

Severe mental illness is associated with shared antenatal and early neurodevelopmental risk, while later on, distinct trajectories convey heterotypic risk for disorders such as schizophrenia or depression (Damme et al., 2022). Epidemiological studies indicate that individuals who are winter- and spring-born have an increased risk of up to 8% to develop schizophrenia (Torrey et al., 1997). In fact, except for known infectious diseases, for no other diseases seasonal birth excesses was described as clearly as those for schizophrenia and bipolar disorder (Torrey et al., 1997). Likewise, an excess of up to 5.5% of depression cases was shown in spring- born subjects (Torrey et al., 1996; Disanto et al., 2012). In addition, one study demonstrated that spring-born individuals had a higher risk of suicidality (Joiner et al., 2002) pointing to an effect of season of birth on this severe and disabling symptom. Although a seasonal birth-excess in psychiatric disorders has been repeatedly reported, the reason for this excess is unclear.

Season of birth acts as a valuable proxy, to study the impact of harmful environmental factors during fetal maturation. Because most infectious agents have seasonal shifts in their incidence, they form a possible explanation for the winter- and spring birth excess in psychiatric disorders. In fact, incidences of prenatal bacterial and viral infections change throughout the year, with a rise during the fall and winter months and decline during spring and summer. Moreover, prenatal infections and inflammation are associated with an elevated risk for psychiatric disorders (Al-Haddad et al., 2019a,b). Early research focused primarily on the identification of pathogens such as toxoplasma gondii, rubella virus, cytomegalovirus, and herpes simplex as possible explanations for the winter–spring birth excess in psychiatric disorders. However, accumulating evidence now suggests that a wide variety of viral and bacterial infections—possibly including COVID-19 (Zaigham and Andersson, 2020; Pantelis et al., 2021) can lead to an increase of psychiatric disorders (see 7 for review). These findings are also in line with the neurodevelopmental hypothesis of schizophrenia (Fatemi and Folsom, 2009) and the fetal-origin hypothesis of mood disorder (Barker, 1998) respectively, suggesting prenatal inflammation to act as a first hit leading to neurodevelopmental abnormalities, which will hamper adaptive brain development and thus increase the risk for major psychiatric disorders.

Depression as well as schizophrenia go along with significant (Shah et al., 2017; Brandl et al., 2019; Hellewell et al., 2019; Huang et al., 2021; Liloia et al., 2021; Serra-Blasco et al., 2021; Gutman et al., 2022), progressive (Vita et al., 2012) transdiagnostic (Goodkind et al., 2015) as well as diagnosis and symptom specific (Horn et al., 2010; Stegmayer et al., 2014, 2016; Walther et al., 2017; Viher et al., 2018; Kindler et al., 2019; Schoretsanitis et al., 2019; Dean et al., 2020; Mertse et al., 2022) patterns of gray matter loss which may represent a hallmark of both disorders. Given the evidence for winter– and spring-birth excesses for schizophrenia, and major depression, and, as already mentioned, the suggested link with neurodevelopmental abnormalities, the question arises as to whether this seasonal birth pattern is associated with alterations in the brain. In fact, animal studies using mouse models of schizophrenia or depression point to alterations in particular within the hippocampus, frontal cortex and the cerebellum following prenatal infections and inflammation (Cotter et al., 1995; Fatemi et al., 1998a,b, 1999, 2002a,b, 2008; Meyer et al., 2008; Ratnayake et al., 2012). This is consistent with the fact that reduced hippocampus volume was confirmed, and alterations within the hippocampus have been largely suggested as relevant for the development of schizophrenia (Nelson et al., 1998; Wright et al., 2000; Heckers, 2001; Honea et al., 2005; Steen et al., 2006; Vita et al., 2006; Heckers and Konradi, 2010; Tamminga et al., 2010) and depression (Sheline et al., 2002; Campbell and MacQueen, 2004; Videbech, 2004; Koolschijn et al., 2009; McKinnon et al., 2009; Cole et al., 2011; Kempton et al., 2011; Bora et al., 2012a,b; Du et al., 2012, 2014; Sacher et al., 2012; Lai, 2013; Sexton et al., 2013; Zhao et al., 2014; Schmaal et al., 2016; Zhang et al., 2016). Effects of season of birth on brain structure were shown in both, depression and schizophrenia. Patients with schizophrenia and depression who are winter-born show a decrease in brain volume, and altered white matter connectivity compared to summer-born patients. In detail, studies confirm ventricular enlargements for winter- and spring-births in schizophrenia (Sacchetti et al., 1987, 1992; Zipursky and Schulz, 1987; Degreef et al., 1988; d’Amato et al., 1994), with some conflicting results (Wilms et al., 1992; Roy et al., 1995). Contrary, summer-born schizophrenia patients had significantly lower fractional anisotropy in widespread white matter regions (i.e., the corpus callosum, internal and external capsule, corona radiata, posterior thalamic radiation, sagittal stratum, and superior longitudinal fasciculus) compared to patients born in the remainder of the year (Giezendanner et al., 2013). Likewise, winter-born patients with bipolar affective depression had more subcortical and periventricular white matter lesions compared to summer-born patients (Moore et al., 2001). Thus, there is evidence of structural alterations in the brain associated with season of birth and schizophrenia as well as depression. However, the distribution of brain alterations as effect of season of birth and whether summer-born or winter-born patients show alterations in brain structure is still unclear. So far, no study assessed transdiagnostic differences in gray matter volume associated with season of birth.

Here we therefore aim to detect effects of season of birth on gray matter volume in a transdiagnostic sample of patients with schizophrenia and depression, compared to healthy controls. Specifically, we hypothesized a relationship between gray matter volume and season of birth in patients with depression as well as patients with schizophrenia and that this association would not be observed in healthy controls. In particular, we suggest decreased volume within the hippocampus in winter-born patients.

## Materials and Methods

### Participants

In total, we included 192 participants, 87 patients with schizophrenia (SZ; 53 winter-born: WB, 34 summer-born: SB), 39 patients with depression (DP; 19 WB; 20 SB), and 66 healthy controls (HC; 42 WB, 24 SB). To stratify participants into seasonal groups, we applied the same cut-off criterion used in previous studies (i.e., winter-born: November through May; summer-born: June through October; Giezendanner et al., 2013). We recruited in- and outpatients at the University Hospital of Psychiatry and Psychotherapy in Bern and healthy controls *via* advertisement and among staff. Patients and controls were the same as in our previous reports (Horn et al., 2010; Walther et al., 2011, Walther et al., 2012a,b; Orosz et al., 2012; Stegmayer et al., 2013, 2014, 2016; Cantisani et al., 2016). Patients were diagnosed according to DSM-IV criteria, while current symptom severity was assessed with the Beck Depression Inventory (Beck, 1961), the Hamilton Depression Inventory (Hamilton, 1986) and the Positive and Negative Syndrome Scale (Kay et al., 1987). Additionally, all participants completed the Mini International Neuropsychiatric Interview (MINI; Sheehan et al., 1998).

Exclusion criteria were substance abuse or dependence (except nicotine), history of head trauma with concurrent loss of consciousness, history of electroconvulsive treatment, a severe medical condition or left-handedness (according to the Edinburgh handedness inventory; Oldfield, 1971). Additional exclusion criteria for controls were history of any psychiatric disorder or a first-degree relative with a schizophrenia spectrum disorder or depression. The local ethics committee (Kantonale Ethikkommission Bern: KEK Bern) approved the study protocol and all participants provided written informed consent.

### Neuroimaging

For structural imaging, a 3D-T1-weighted Modified Driven Equilibrium Fourier Transform Pulse (MDEFT) Sequence (Deichmann et al., 2004) was acquired on a 3-T Siemens Magnetom TrioTim Scanner System, equipped with a standard 12-channel radio frequency head coil (Siemens Vision, Erlangen, Germany). This sequence provided 176 sagittal slices with 256 × 256 matrix points, a 256 × 256 field of view (FOV), and a nominal isotopic resolution of 1 mm × 1 mm × 1 mm. Further scan parameters were 7.92 ms repetition time (TR), 2.48 ms echo time (TE) and a flip angle (FA) of 16°. We preprocessed all resulting high-resolution images with the SPM 12 (Ashburner and Friston, 2000; Wellcome Trust Center for Neuroimaging, London^1^). All preprocessing steps were conducted using standard procedures as implemented in SPM 12, in particular the voxel-based morphometry (VBM) toolbox. Default settings were used. The images have been normalized and modulated. Structural images were bias-corrected, tissue-classified and normalized to Montreal Neurological Institute space using linear (12-parameter affine) and non-linear transformations. Gray matter volume per voxel was calculated by applying an absolute threshold masking of 0.1 and modulating the normalized segmented images with a non-linear only warping. For quality check of the procedures, the normalized, bias-corrected images were visually inspected. MRI images with artifacts, anatomical abnormalities as well as neurodegenerative changes were excluded. Finally the normalized, segmented and modulated volumes were smoothed with an 8 mm full width at half maximum (FWHM) Gaussian kernel (Honea et al., 2005).

### Statistical Analyses

We analyzed structural images with SPM 12 (Wellcome Trust Centre for NeuroImaging, University College London, United Kingdom) and demographic and clinical data with SPSS for windows (IBM, version 26.0). Univariate analyses, two-sample *t*-tests and chi-square tests (χ*^2^*) were used, respectively.

Our main investigative interest was the effect of patient status (SZ, DP, HC) and season of birth (WB, SB) on whole brain gray matter volume. We therefore performed a one-way analysis of covariation (ANCOVA) over six groups (SZ_*WB*_, SZ_*SB*_, DP_*WB*_, DP_*SB*_, HC_*WB*_, and HC_*SB*_). To control for trend-level gender differences between summer- and winter-born subjects as well as variability in head sizes, we added total gray matter volume and gender as covariates into the main model. We then performed an outlier-analyses of the ANCOVA over the six groups extracting gray matter values of the significant clusters. We considered a value to be an outlier, if it lied either below or above the following ranges: The 1st quartile—1.5 × interquartile range, or the 3rd quartile + 1.5 × interquartile range. Three potential outlier were identified and subsequently removed from all further analyses—two within the schizophrenia- summer-born group and one within the healthy control summer-born group. No outlier were identified within either of the depression groups. Furthermore, as patient groups differed in education and age, we provide the results of the whole-brain ANCOVA with education and age as additional covariates of no interest in the Supplementary Material.

In addition, we plotted extracted mean gray matter values for the six groups and performed *post hoc* comparisons of extracted GM values between patient groups and summer- and winter-born subjects applying univariate analyses and *t*-tests, respectively. To examine a group × season interaction effect on hippocampal volume, we calculated a two-way ANOVA on hippocampal volume [with factors season of birth (SB vs WB) and patient status (SZ vs HC vs DP)] (Supplementary Table 4 and Supplementary Figure 3). We corrected *post hoc* group comparisons with Sidak-correction for multiple testing.

Finally, we assessed the season-of-birth effect on whole brain GM volume for the patient status groups separately (DP, SZ and HC). We therefore performed *t*-tests within the ANCOVA, comparing mean gray matter values of WB and SB individuals within SZ, DP and HC participants, respectively. We report imaging results yielding significance at *p* < 0.05 (FWE-corrected). For illustration purposes, all images are displayed at a threshold of *p* < 0.001, cluster sizes *k* > 50 voxels, uncorrected. To provide additional information, we show the results of the whole brain contrasts at a lower threshold (*p* < 0.001 uncorrected, cluster sizes *k* > 50 voxels) in the Supplementary Material. We calculated effect sizes for *F*-tests: η*_*p*_^2^* (eta^2^), based on *F*-value, df-1 and df-2, for *t*-tests: *d* (Cohen’s D), based on *t*-value and df and for χ*^2^*-test: φ (phi) calculated based on χ^2^-value, and sample size.

## Results

WB patients with schizophrenia and depression, as well as WB healthy controls, did not differ in age, gender and education from their SB counterparts. Likewise, WB subjects over all groups did not differ significantly in age and education from SB individuals. As expected, patients with schizophrenia included more male and patients with depression more female participants. In addition, patients with depression were older than patients with schizophrenia. Demographic and clinical variables are provided in Table 1.

**TABLE 1:** Demographic and clinical variables.

Schizophrenia		WB (*n* = 53)	SB (*n* = 34)	*df*	*t/X^2^*	*p*	*d/φ*
Age (M/SD)		37.6 (11.1)	34.9 (11.4)	85	1.086	0.281	0.235
Gender (male%)		30(56.6%)	23(67.6%)	1	1.061	0.303	0.112
Education (M/SD)		13.2 (3.8)	13.0 (3.0)	85	0.244	0.808	0.053
Nr. of episodes (M/SD)		4.8 (5.2)	6.5 (7.9)	85	–1.230	0.222	–0.267
PANSS total (M/SD)		64.4 (18.3)	68.2 (18.4)	85	–0.950	0.345	–0.206
PANSS pos (M/SD)		15.9 (6.5)	17.7 (6.6)	85	–1.281	0.204	–0.277
PANSS neg (M/SD)		16.5 (6.3)	16.8 (5.8)	85	–0.198	0.843	–0.043
CPZ (M/SD)		429.5 (342.5)	416.4 (347.0)	85	0.173	0.863	0.038

**Depression**		**WB (*n* = 19)**	**SB (*n* = 20)**	***df***	***t/X^2^***	***p***	***d/φ***

Age (M/SD)		44.4 (11.4)	43.5 (14.5)	37	0.207	0.837	0.068
Gender (male%)		6(31.6%)	10(50.0%)	1	1.367	0.242	0.189
Education (M/SD)		15.5 (5.5)	13.6 (2.5)	37	1.373	0.178	0.451
Nr. of episodes (M/SD)		8.3 (9.1)	12.2 (22.3)	37	–0.716	0.479	–0.235
HAMD total (M/SD)		22.6 (7.4)	24.3 (5.6)	37	–0.765	0.450	–0.251
BDI total (M/SD)		22.0 (11.1)	27.2 (10.9)	37	–1.425	0.163	–0.469

**Healthy Controls**		**WB (*n* = 42**)	**SB (*n* = 24)**	***df***	***t/X^2^***	***p***	***d/φ***

Age (M/SD)		40.3 (15.2)	37.3 (12.9)	64	0.802	0.426	0.201
Gender (male%)		21(50.0%)	16(66.7%)	1	1.722	0.189	0.160
Education (M/SD)		14.8 (3.3)	14.0 (2.8)	64	0.960	0.341	0.240

**All subjects**		**WB (*n* = 114)**	**SB (*n* = 78)**	***df***	***t/X*^2^**	***p***	***d/φ***

Age		39.7 (12.9)	37.9 (13.0)	190	0.975	0.331	0.143
Gender (male%)		57(50.0%)	49(62.8%)	1	3.078	0.079	0.127
Education		14.2 (4.0)	13.5 (2.8)	190	1.308	0.192	0.190

**Patient Status Groups**	**SZ (*n* = 87)**	**DP (*n* = 39)**	**HC (*n* = 66)**	***df***	***F/X^2^***	***p***	***d/φ***

Age	36.57 (11.25)	43.92 (12.91)	39.21 (14.35)	2	4.508	0.012	0.046
Gender (male%)	53(60.9%)	16(41.0%)	37(56.1%)	2	4.339	0.114	0.150
Education	13.14 (3.5)	14.51 (4.3)	14.48 (3.1)	2	3.467	0.033	0.035

*WB = winter-born; SB = summer-born; SZ = schizophrenia; DP = depression; HC = healthy controls; PANSS = Positive and Negative Syndrome Scale; pos = positive symptoms; neg = negative symptoms; HAMD = Hamilton rating scale for depression; BDI = Beck Depression Inventory; CPZ = Chlorpromazine equivalent dosage; df = degrees of freedom; M = Mean; SD = Standard deviation; IQR = interquartile range; X^2^ = Chi-squared test.*

### Lower Hippocampal Volume in Summer-Born Patients With Depression

**FIGURE 1:**
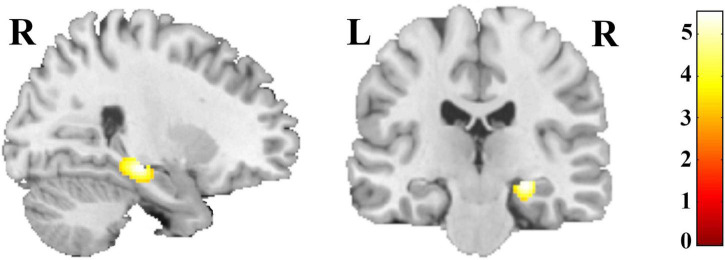
Regions with differences in GM volume in summer- and winter-born patients with depression, schizophrenia and healthy controls; for illustration purpose threshold was set at *p* < 0.001, cluster sizes: *k* > 50 voxels; uncorrected.

The whole-brain analysis revealed a group effect within the right hippocampus, pFWE-corr = 0.015; F (Joiner et al., 2002) = 8.47; η_*p*_^2^ = 0.190; k = 11 voxels; x = 26, y = −24, z = −12 (Figure 1). Lowering the threshold (*p* < 0.001, cluster sizes: *k* > 50 voxels; uncorrected) revealed additional frontal and orbital clusters (see Supplementary MaterialSupplementary Figure 1 and Supplementary Table 1). Including education and age as additional covariates yielded substantially the same results (see Supplementary Table 2).

**FIGURE 2:**
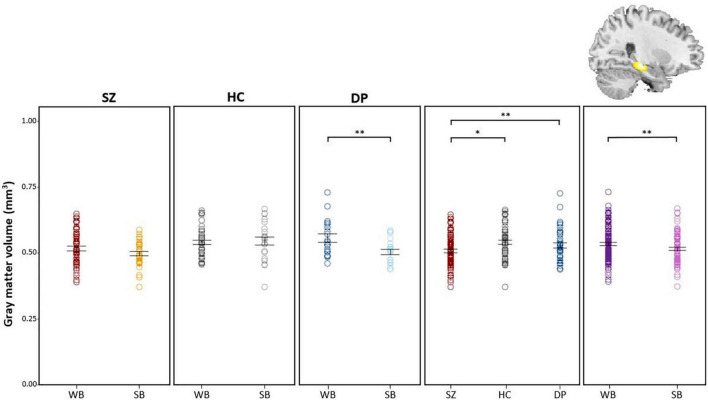
*Post hoc* comparisons of extracted GM values show decreased volume of the hippocampus in DP_*SB*_ vs. DP_*WB*_ patients, in SZ patients vs. HC participants, in SZ patients vs DP patients and in WB vs SB individuals. *WB* = Winter-born; *SB* = Summer-born; *SZ* = Schizophrenia; *DP* = Depression; *HC* = Healthy Control; ^**^p < 0.01, *p < 0.05.

*Post hoc* comparisons of extracted gray-matter values showed a decrease of right hippocampal volume in summer-born DP, when compared to winter-born DP. Furthermore, SZ patients had a decreased hippocampal volume compared to HC participants and DP patients, independent of season of birth (Figure 2). Finally, summer-born subjects, independent of group showed lower hippocampal volume compared to winter-born subjects.

Likewise whole brain *t*-tests within the ANCOVA revealed a decreased volume within the hippocampus in DP_*SB*_ (*_p_*_FWE–corr_ = 0.003; *t* = 5.48; x = 26, y = −22, z = −12) (Supplementary Figure 2 and Supplementary Table 3). We detected no significant differences comparing SZ_*SB*_ and SZ_*WB*_ patients.

## Discussion

Here we test the effect of season of birth on gray matter volume in a large transdiagnostic sample of 192 subjects with depression, schizophrenia and healthy controls. As hypothesized, we demonstrate a significant effect of season of birth on gray matter volume. In particular, we found an association of season of birth and right hippocampal volume in depression. However, contrary to our hypothesis summer-born patients showed decreased hippocampal volume compared to winter-born patients with depression. No effect of season of birth was present in schizophrenia. In contrast, schizophrenia patients had a right hippocampal volume reduction independent of season of birth.

Volumetric changes shown with VBM cannot offer direct information about the underlying cellular mechanism relevant for the effects. Therefore, deductive reasoning from volumetric changes to functional changes remains speculative. However, it has been suggested that volumetric changes seen with VBM are the result of a multifactorial process including multiple cellular modifications, for example cell density, cell size, myelination and vascularization affecting relaxation times and voxel intensities on a T1-weighted image (Zatorre et al., 2012). Given that season of birth can serve as a proxy of harmful environmental factors, our results argue for such environmental factors leading to hippocampal volume reduction in summer-born depression.

As stated in the introduction, a birth excess in winter-born patients with depression has repeatedly been found. In fact, harmful environmental factors are thought to affect neurodevelopment in perinatal stages and thus increase the risk to develop the disorder. Importantly previous reports suggest harmful effects of season of birth in depression. According to these observations we expected a decreased gray matter volume, in particular within the hippocampus in winter-born patients. Previous reports show alterations in hippocampal development leading to reduction in gray matter within the hippocampus following prenatal infection. For instance, reduced cell density in pyramidal and non-pyramidal cells as well as signs of atrophy (e.g., Fatemi et al., 1999, 2002a,^2008^) were demonstrated. These alterations were associated with inflammation-induced depressive-like behavior in mice such as decrease of exploratory behavior (Shi et al., 2003; Meyer, 2006; Samuelsson et al., 2006; Fortier et al., 2007; Meyer et al., 2008; Li et al., 2009; Spini et al., 2021). On the contrary, here we show gray matter reduction in the hippocampus in summer-born compared to winter-born patients. Therefore, we have to conclude that the described seasonal birth excess in winter is not related to hippocampal volume reduction in depression.

The hippocampus is one of the most studied brain regions in the context of depression. In fact, bilateral hippocampal volume reductions form the most reliable regional gray matter abnormalities identified in depression (Koolschijn et al., 2009; McKinnon et al., 2009; Cole et al., 2011; Kempton et al., 2011; Bora et al., 2012a,b; Du et al., 2012, 2014; Sacher et al., 2012; Arnone et al., 2013; Lai, 2013; Sexton et al., 2013; Stratmann et al., 2014; Zhao et al., 2014; Schmaal et al., 2016; Zhang et al., 2016; Yüksel et al., 2018) and subjects with subclinical symptoms (Besteher et al., 2020). The hippocampus is a brain region specifically sensitive to infectious agents (Green and Nolan, 2014), is important for stress regulation for instance *via* its inhibitory control over HPA-axis activity, and is more broadly involved in cognitive and affective processing *via* its widespread connections with prefrontal and limbic brain regions (Duman and Monteggia, 2006). Furthermore, antidepressant action may be accomplished through the prevention of cell apoptosis in the hippocampus (McKernan et al., 2009; Chen et al., 2017). Likewise, modern models of depression suggest hippocampal atrophy in humans as key in the development of the disease. In fact, lower hippocampal volume has been suggested as a risk marker of depression (Chen et al., 2010; Rao et al., 2010; Cole et al., 2011). Reduced hippocampal volume has been consistently shown to be about 5% smaller in depression (for meta-analyses see Koolschijn et al., 2009; McKinnon et al., 2009; Cole et al., 2011; Kempton et al., 2011; Bora et al., 2012a,b; Du et al., 2012, 2014; Sacher et al., 2012; Arnone et al., 2013; Lai, 2013; Sexton et al., 2013; Zhao et al., 2014; Schmaal et al., 2016; Zhang et al., 2016). Importantly, reductions in hippocampal volume are not only explained as consequence of medication (Zhao et al., 2014) or psychiatric comorbidities (Du et al., 2012) and have been shown throughout the lifespan (Sexton et al., 2013). Thus, reductions in hippocampal volume are a robust structural marker observed in depression. Our finding of reduced hippocampal volume in summer-born patients with depression add to this evidence and suggesting a key role of environmental factors as perinatal events in the disturbance of hippocampal development as risk factor for depression leastwise in a subgroup of patients. Importantly although previously alterations associated with season of birth in summer-born patients were not shown in depression, this effect was seen in schizophrenia and healthy subjects with psychotic experiences. In detail, summer-born schizophrenia patients had significantly lower fractional anisotropy in widespread white matter regions (i.e., the corpus callosum, internal and external capsule, corona radiata, posterior thalamic radiation, sagittal stratum, and superior longitudinal fasciculus) compared to patients born in the remainder of the year (Kempton et al., 2011), and cortical cortical thinning was detected in summer-born healthy individuals with subthreshold psychosis symptoms (Koolschijn et al., 2009).

To the best of our knowledge, this is the first study to investigate the effect of season of birth on gray matter volume in a transdiagnostic sample of patients, in particular including depression. The mechanisms that account for the detected reduced hippocampal volume in depressed summer-born patients are beyond the scope of this study and understanding the pathophysiological mechanisms by which infection, inflammation, and depression are linked is complex. It was hypothesized that several factors (for instance increased oxidative stress, hypothalamic–pituitary–adrenal axis dysfunction, neurotransmitter insufficiency or reductions in growth factors) or a combination of these factors may lead to a possible final pathway of decreases in neuropil, immunoreactivity, and dendritic spine density or neuronal apoptosis that may underlay gray matter loss in the hippocampus. However, several other factors such as unhealthy lifestyle in patients may also contribute to our finding. Still, the present finding of a relationship between season of birth and right hippocampal volume support the hypothesis of perinatal events involving a seasonal factor and subsequent pathologic brain development in depression.

Turning to schizophrenia, we show a general reduction of hippocampal volume irrespective of season of birth. This fact may hamper to detect specific effects of season of birth within the hippocampus. However, our finding is in line with previous reports showing hippocampal volume reduction in schizophrenia (Nelson et al., 1998; Heckers and Konradi, 2010; Adriano et al., 2012), even at the onset of the disorder in the first episode (Adriano et al., 2012; McHugo et al., 2020), as well as in ad risk subjects who later develop the disorder (Harrisberger et al., 2016). Contrary, and as mentioned in the introduction, previous reports have observed alterations in the brain structure associated with season of birth, which was not the case in our report. In particular, ventricular enlargement in winter-born compared to summer-born patients with schizophrenia was shown (Sacchetti et al., 1987, 1992; Zipursky and Schulz, 1987; Degreef et al., 1988; d’Amato et al., 1994). In addition, one DTI study displayed structural white matter impairments in patients born in summer relative to patients born in winter (Giezendanner et al., 2013).

We have to point out limitations of our report. First, all but seven patients were on psychotropic medication. We cannot rule out that medication had an effect on hippocampal volume. In particular, medication may hamper to detect an effect of volume reduction in schizophrenia. However, SB and WB schizophrenia patients did not differ in CPZ equivalents as a proxy of antipsychotic dosage. In addition, previous reports detected alterations in brain structure in medicated schizophrenia patients (Sacchetti et al., 1987, 1992; Zipursky and Schulz, 1987; Degreef et al., 1988; d’Amato et al., 1994; Giezendanner et al., 2013). Furthermore meta-analytic evidence suggests that hippocampal volume reduction in depression is not solely explained as a medication effect (Zhao et al., 2014). Second, we do not have information of possible infections or other complications during the prenatal period or birth as well as birth weight or whether it was preterm birth in our subjects. Thus, the seasonal birth pattern is associated with alterations in the brain but we cannot conclude on the causes. Third, sample size was not balanced over the groups leading to smaller sample size of patients with depression (*n* = 39). Finally, several factors have been suggested that may moderate the association between depression and hippocampal volume including depression severity and state (Arnone et al., 2013) or age-of-onset of the first depressive episode (Schmaal et al., 2016). However, in our study winter- and summer-born patients with depression did not differ in depression severity and age of onset.

In conclusion, our results demonstrate that seasonal birth pattern may contribute to hippocampal volume reduction in depression. Additionally, we demonstrate that schizophrenia patients show a hippocampal volume reduction independent of season of birth. The finding of a relationship between season of birth and hippocampal volume in depression support the hypothesis of a harmful perinatal event and subsequent pathologic brain development in depression, at least in a subgroup of patients.

## Data Availability Statement

The raw data supporting the conclusions of this article will be made available by the authors, without undue reservation.

## Ethics Statement

The studies involving human participants were reviewed and approved by Ethics Commission of the Canton of Bern [Kantonale Ethikkomission Bern (KEK)], Switzerland. The patients/participants provided their written informed consent to participate in this study.

## Author Contributions

SW designed the study, wrote the protocol, acquired the funding, and supervised the data acquisition. KS analyzed the data and wrote the first draft of the manuscript. NS, NA, and FC recruited the subjects and conducted the assessments. All authors discussed findings and edited the manuscript.

## Conflict of Interest

SW received honoraria from Janssen, Lundbeck, Mepha, Neurolite, Otsuka and Sunovion, and served on advisory boards for Lundbeck and Sunovion in 2019. KS received honoraria from Janssen, Lundbeck, Mepha, and Sunovion. RH received speaker/advisor honorary from Merck, Novartis, Roche, Biogen, Alexion, Sanofi, Janssen, Bristol-Myers Squibb, and Almirall, and received research support within the last 5 years from Roche, Merck, Sanofi, Biogen, Chiesi, and Bristol-Myers Squibb, and also received research grants from the Swiss MS Society, and also serves as associated editor for Journal of Central Nervous System disease. The remaining authors declare that the research was conducted in the absence of any commercial or financial relationships that could be construed as a potential conflict of interest.

## Publisher’s Note

All claims expressed in this article are solely those of the authors and do not necessarily represent those of their affiliated organizations, or those of the publisher, the editors and the reviewers. Any product that may be evaluated in this article, or claim that may be made by its manufacturer, is not guaranteed or endorsed by the publisher.

## Footnotes

1
http://www.fil.ion.ucl.ac.uk/spm
