# Supplementary material for: Effect of Season of Birth on Hippocampus Volume in a Transdiagnostic Sample of Patients With Depression and Schizophrenia
Source: Front Hum Neurosci. 2022 Jun 13;16:877461. doi: 10.3389/fnhum.2022.877461 (PMC9234120; doi:10.3389/fnhum.2022.877461)
Supplement: Supplementary file 1 [file Data_Sheet_1.docx]

Supplementary Material

*
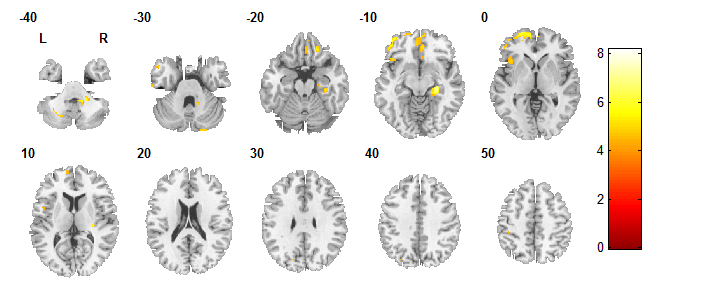
*

# Figure S1. Additional clusters after whole-brain analysis with lower threshold. Whole brain one-way analysis of covariation (ANCOVA) between all participant groups (SZ_WB_, SZ_SB_, DP_WB_, DP_SB_, HC_WB_, HC_SB_). Gray matter volume differences are displayed at *p* < .001, *k* > 50 voxels, uncorrected.

# Table S1. Additional clusters after whole-brain analyses with lower threshold.

|  | MNI Coordinates | | |  | |  |  |
| --- | --- | --- | --- | --- | --- | --- | --- |
| Regions | X | Y | Z | #voxels | *P*_(FWE-corr)_ | *F*(5, 181) | *η_p_^2^* |
| R Hippocampus | 26 | -24 | -12 | 279 | .015 | 8.47 | .190 |
| L lateral orbital gyrus, L orbital inferior frontal gyrus, L middle frontal gyrus | -42 | 44 | -12 | 116 | .705 | 5.87 | .140 |
| L medial frontal gyrus, L superior frontal gyrus, gyrus rectus, middle orbital gyrus | -22 | 62 | 0 | 157 | .881 | 5.52 | .132 |
| R Cerebellum | 14 | -42 | -40 | 60 | .892 | 5.50 | .132 |
| R Orbitofrontal Gyrus | 6 | 26 | -14 | 87 | .990 | 5.05 | .122 |

Whole brain one-way analysis of covariation (ANCOVA) between all participant groups (SZ_WB_, SZ_SB_, DP_WB_, DP_SB_, HC_WB_, HC_SB_), including total gray matter and gender as covariates of no interest. Gray matter volume differences are displayed at *p* < .001, *k* > 50 voxels, uncorrected. *Abbreviations*: FWE-corr = family-wise error corrected; R = right; L = left.

# Table S2. Whole-brain analyses of covariation, with education and age as additional covariate of no interest.

|  | MNI Coordinates | | |  | |  |  |
| --- | --- | --- | --- | --- | --- | --- | --- |
| Regions | X | Y | Z | #voxels | *P*_(FWE-corr)_ | *F*(5, 181) | *η_p_^2^* |
| R Hippocampus | 26 | -24 | -12 | 114 | .182 | 6.96 | .161 |
| L Insula, inferior frontal gyrus | -42 | 22 | -8 | 60 | .544 | 6.14 | .145 |
| R Cerebellum | 10 | -52 | -28 | 153 | .600 | 6.05 | .143 |
| L inferior frontal gyrus | -48 | 40 | 2 | 93 | .926 | 5.41 | .130 |
| L middle frontal gyrus, L superior frontal gyrus | -28 | 60 | 4 | 69 | .978 | 5.17 | .125 |

Whole-brain one-way analysis of covariation (ANCOVA) between all participant groups (SZ_WB_, SZ_SB_, DP_WB_, DP_SB_, HC_WB_, HC_SB_), including gender, total gray matter volume, age, and education as covariates of no interest. Gray matter volume differences are displayed at *p* < .001, cluster sizes: *k* > 50 voxels, uncorrected. *Abbreviations*: FWE-corr = family-wise error corrected; R = right; L = left.


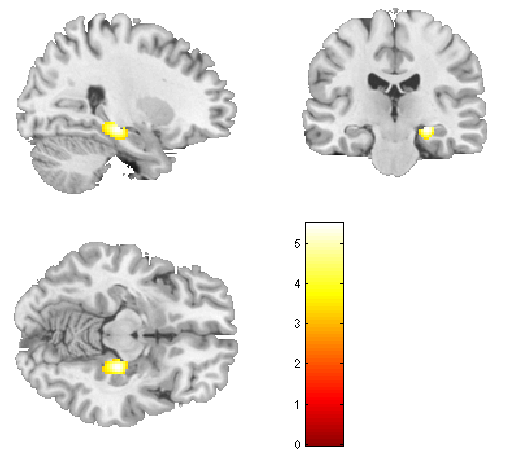


# Figure S2. Decreased hippocampal volume in summer-born (vs. winter-born) patients with depression. Whole brain *t*-tests within the analysis of covariation (ANCOVA) between summer- and winter-born patients with depression (*p*_FEW-corr_ = .003; *t* = 5.48 *k* = 255; x = 26, y = -22, z = -12). Covariates of no interest included: gray matter volume, and gender.

**Table S3.** Whole brain *t*-test comparisons between summer-born and winter-born individuals.

|  | MNI Coordinates | | |  | |  |  |
| --- | --- | --- | --- | --- | --- | --- | --- |
|  | X | Y | Z | #voxels | *P*_(FWE-corr)_ | *t (df)* | *d* |
| DP |  |  |  |  |  |  |  |
| R Hippocampus | 26 | -22 | -12 | 255 | .003 | 5.48 (37) | 1.80 |
| HC |  |  |  |  |  |  |  |
| R/L Cerebellum | 18 | -42 | -40 | 1341 | .015 | 5.08 (63) | 1.28 |
| R Hippocampus | 38 | -4 | -24 | 83 | .073 | 4.68 (63) | 1.18 |
| L superior frontal gyrus, medial frontal gyrus, middle orbital gyrus | -16 | 58 | 0 | 169 | .265 | 4.29 (63) | 1.08 |
| L inferior frontal gyrus | -52 | 26 | 10 | 98 | .372 | 4.17 (63) | 1.05 |
| L superior temporal gyrus, medial temporal gyrus, inferior temporal gyrus | -40 | 2 | -28 | 356 | .485 | 4.06 (63) | 1.02 |
| L insula, inferior frontal gyrus, precentral gyrus | -42 | 6 | 16 | 236 | .503 | 4.04 (63) | 1.02 |
| SZ |  |  |  |  |  |  |  |
| R Insula | 30 | -22 | 12 | 62 | .796 | 3.78 (83) | 0.95 |

Whole brain *t*-test comparisons were performed within the analysis of covariation (ANCOVA), between summer- and winter-born individuals of all participant status groups (DP, SZ, and HC). *Abbreviations*: DP = patients with depression; SZ = patients with schizophrenia; HC = healthy controls; FWE-corr = family-wise error corrected; R = right; L = left. Covariates of no interest included: gray matter volume, and gender.

**Table S4**. Group x season interaction effect of right hippocampal gray matter values.

|  | *F (df-1, df-2)* | *p* | *η_p_^2^* |
| --- | --- | --- | --- |
| Group (SZ, DP, HC) | 7.18 (2, 181) | .001 | .073 |
| Season of birth (WB, SB) | 18.74 (1, 181) | .000 | .094 |
| Group x Season of birth | 3.63 (2, 181) | .028 | .039 |

Two-way ANCOVA of extracted gray matter values revealed a group (SZ vs HC vs DP) x Season

(SB vs WB) interaction effect. *Abbreviations:* DP = patients with depression; SZ = patients with schizophrenia; HC = healthy controls; WB = winter-born; SB = summer-born. Covariates of no interest included: gray matter volume, and gender.


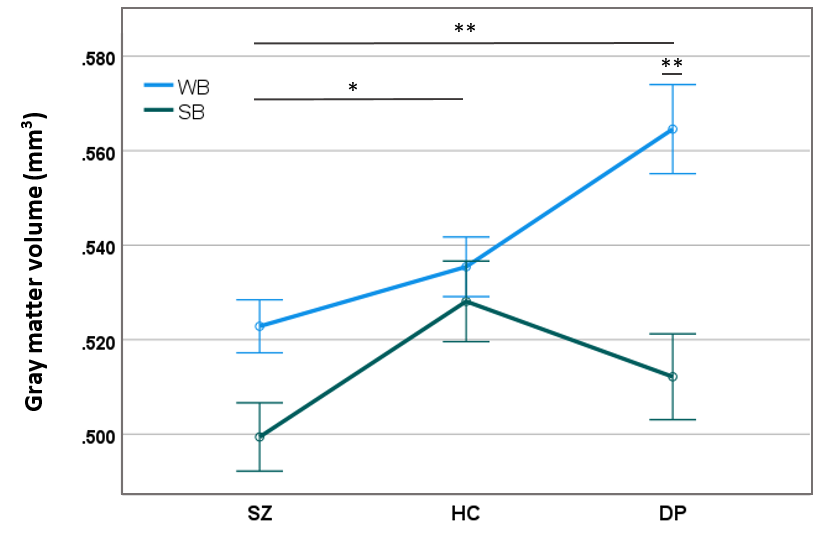


**Figure S3.** Group (SZ vs HC vs DP) x Season (WB vs SB) interaction effect of extracted hippocampal gray matter values. SZ_WB_ = .523 (.006), SZ_SB_ = .499 (.007), HC_WB_ = .535 (.006), HC_SB_ = .528 (.009), DP_WB_ = .565 (.009), DP_SB_ = .512 (.009); **p < .01, * p < .05; Post-hoc comparisons between groups are Sidak-corrected for multiple testing; *Abbreviations*: DP = patients with depression; SZ = patients with schizophrenia; HC = healthy controls; WB = winter-born; SB = summer-born. Means are corrected for total gray matter volume and gender
